# Reactivity of Heteropolymolybdates and Heteropolytungstates in the Cationic Polymerization of Styrene

**DOI:** 10.3390/molecules15053319

**Published:** 2010-05-05

**Authors:** Ahmed Aouissi, Zeid Abdullah Al-Othman, Holeil Al-Anezi

**Affiliations:** Chemistry Department, College of Science, King Saud University, Riyadh, Saudi Arabia

**Keywords:** heteropoly compounds, cationic polymerization, polystyrene, solid acid

## Abstract

The two heteropolyacids H_3_PW_12_O_40_ and H_3_PMo_12_O_40_, and their homologous salts (NH_4_)_3_PW_12_O_40_, and (NH_4_)_3_PMo_12_O_40_ were prepared and tested in the cationic polymerization of styrene. The results showed that the heteropolytungstates were more reactive than the heteropolymolybdates. It has been found that the yield and the viscosity average molecular weight (M_v_) of polystyrene are directly proportional to the acidity strength of the heteropolyanions (H_3_PW_12_O_40_ > H_3_PMo_12_O_40_ > (NH_4_)_3_PW_12_O_40_ > (NH_4_)_3_PMo_12_O_40_). The highest yield (68.0%) and M_v_ (7,930) were obtained by using H_3_PW_12_O_40_. In addition, H_3_PW_12_O_40_ polymerized the styrene under mild conditions and was recyclable, and could behave as a truly heterogeneous catalyst.

## 1. Introduction

Modern chemical industry necessitates much more efficient usage of energy and resources, in order to minimize the undesirable environmental impact, competing with the rapidly growing population. In this context solid acid catalysts have attracted much attention in organic synthesis owing to their easy work-up procedures, easy filtration, and minimization of cost and waste generation due to reuse and recycling of these compounds [1]. The advantages of heterogeneous catalysts over homogeneous ones include stability (towards air and moisture), lack of corrosion, ease of handling, recovery and regeneration. Among the heterogeneous catalysts used, numerous works have reported the application of heteropolyanions as new materials in various fields.

Heteropolyanions are oxoclusters of transition metals such as W and Mo. They are discrete species, insoluble in non-polar solvents but highly soluble in polar ones without structure change. Due to their unique combination of acid-base and redox properties these kinds of solids have been used successfully as solid catalysts in their acidic or their salt form for acid and redox catalyzed reactions in both homogeneous and heterogeneous media [2,3,4,5,6,7]. Because of their stronger acidity, they generally exhibit higher catalytic activity than conventional acids, such as sulfuric and nitric acids [8,9,10]. Recently heteropolyanions had been introduced into the field of polymer science [11,12,13,14,15]. Chen *et al*. [11] have studied the polymerization of styrene over 12-molybdophosphoric acid. To the best knowledge of the authors, other heteropolyanions with much stronger acidity, namely 12-tungstophosphoric acid were not investigated. Thus, in this paper, the investigation was extended to a series of heteropoly compounds having W and Mo as added atoms and we have attempted to compare the reactivity between W and Mo in the cationic polymerization of styrene. It is worth noting that Mo and W heteropolyoxoanions have attracted much attention in the area of catalytic reactions [3,12].

## 2. Results and Discussion

### 2.1. Catalyst characterization

#### 2.1.1. Infrared spectra

**Figure 1:**
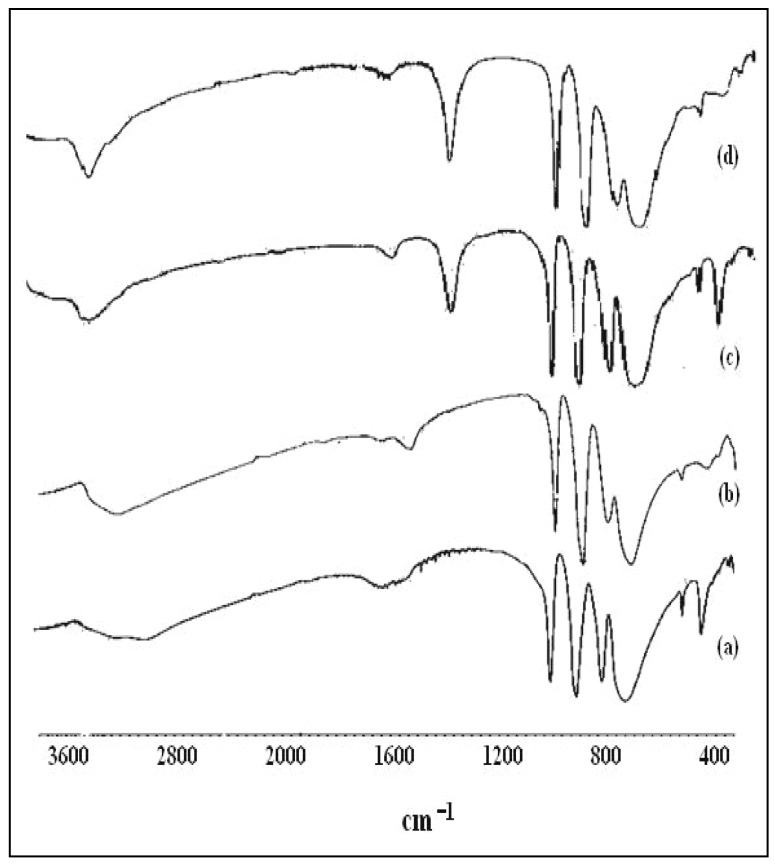
IR spectra of: a) H_3_PW_12_O_40_; b) H_3_PMo_12_O_40_; c) (NH_4_)_3_PW_12_O_40_; d) (NH_4_)_3_PMo_12_O_40_.

The spectra of the catalysts used are shown in Figure 1. The IR spectra have been assigned according to [16,17]. The main characteristic features of the Keggin structure are observed at 1,080–1,060 cm^-1^ (ν_as_ P-O_a_), at 990–960 cm^-1^ (ν_as_ M-O_d_), at 900–870 cm^-1^(ν_a_ M-O_d_-M), and at 810–760 cm^-1^ (ν_as_ M-O_c_-M), respectively (M = W or Mo). 

Besides the above characteristic frequencies of the heteropolyanions, in spectra c) and d) there is a broad band at 3,212 cm^-1^, and a sharp band at 1,414 cm^-1^, which are characteristic absorptions of the NH_4_^+^ group, which indicates the formation of the ammonium salts (NH_4_)_3_PW_12_O_40_ and NH_4_)_3_PMo_12_O_40_ [16].

#### 2.1.2. Characterization of the polymer product

**Figure 2:**
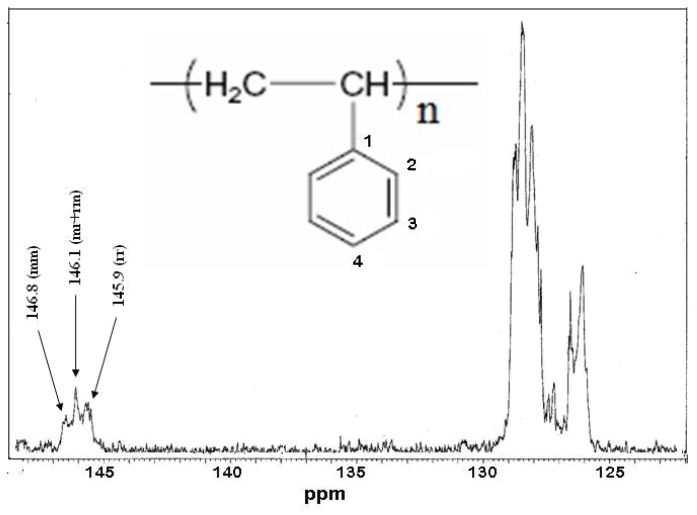
100 MHz ^13^C-NMR spectrum in CDCl_3_ of polystyrene obtained with H_3_PW_12_O_40_ catalyst.

The results showed that these four solid acid catalysts induced the polymerization of styrene, as evidenced by ^13^C-NMR (Figure 2). 

The analysis of the ^13^C-NMR spectra showed the signals of aliphatic carbon atoms at 40.4 and 43.8 ppm and signals corresponding to the *meta*, *ortho* and *para* carbon atoms of the aromatic ring in the region between 125.5 and 128.0 ppm. The aromatic C_1_-carbons gave three signals at 145.3, 145.7 and 146.1 ppm attributed to the (rr), (mr + rm) and (mm) diads, respectively, according to [18]. It was found that this kind of catalyst essentially gave a mixture of diads (mr + rm) (65–70%), rr (20–25%) and a small quantity of diad mm (10–15%).

**Figure 3:**
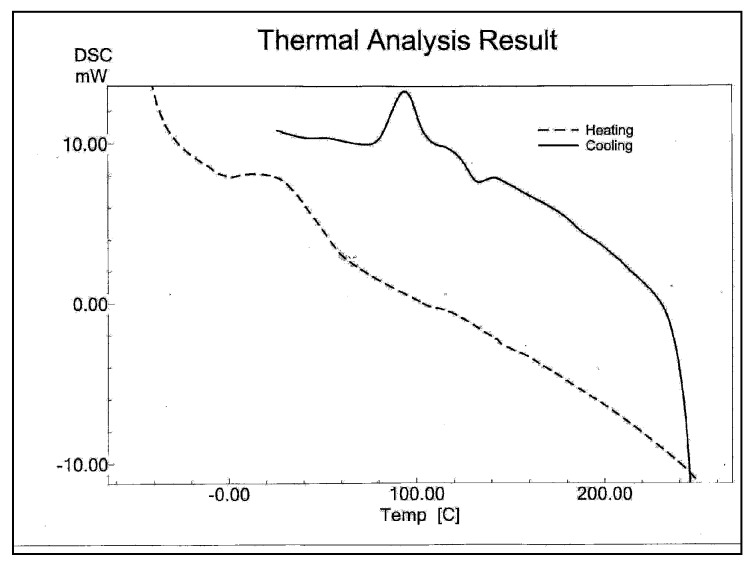
The DSC thermograms of polystyrene material obtained at 20 °C/min heating and cooling rate.

Differential scanning calorimetry studies characterized further these polystyrene materials. Figure 3 shows a glass transition temperature (T_g_), melting temperature (T_m_) and crystallization temperature (T_c_) at 58.2 °C, 149.9 °C, and 94.5 °C respectively.

### 2.2. Catalyst reactivity

#### 2.2.1. Effect of the styrene/DCM ratio

**Figure 4:**
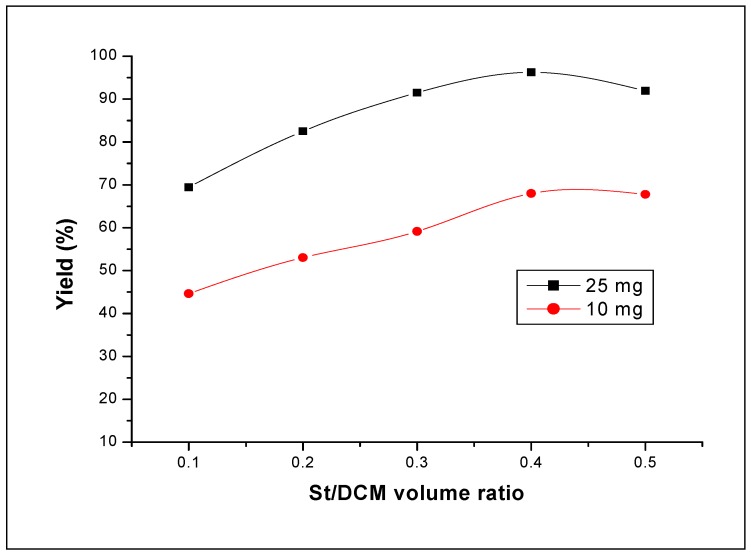
Effect of St/DCM volume ratio on styrene polymerization using H_3_PW_12_O_40_ catalyst. Reaction conditions: T = 25 °C; catalyst amount 10 mg and 25 mg; reaction time 160 min.

Since the bulk polymerization of styrene was rapidly stopped and the magnetic bar could not stir, we decided to perform the polymerization reactions in a solvent. The styrene polymerization reactions were thus carried out in dichloromethane (DCM) in the presence of H_3_PW_12_O_40_ as catalyst in order to examine the effect of the solvent ratio on the catalytic properties of the catalyst and therefore select the suitable St/solvent ratio. The polymerization reactions were catalyzed by 10 mg and 25 mg of H_3_PW_12_O_40_. The results are shown in Figure 4. It can be seen from this figure that for both catalyst amounts, the best yield was obtained for the volume ratio St/DCM = 0.4.

#### 2.2.2. Reactivity of W and Mo in the heteropolyacids

**Figure 5:**
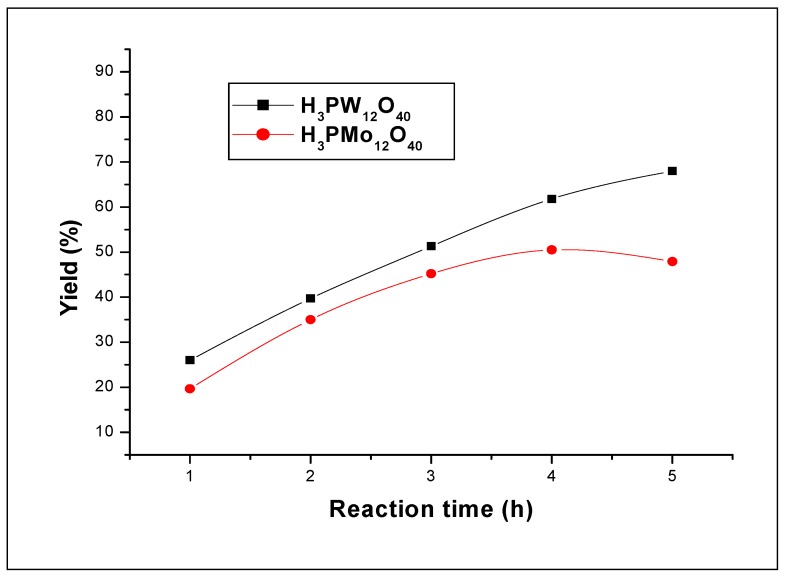
Effect of the reaction time on the styrene polymerization (in dichloromethane) over H_3_PW_12_O_40_ and H_3_PMo_12_O_40_ catalysts. Polymerization was carried out at 25 °C using 10 mg of catalyst.

**Figure 6:**
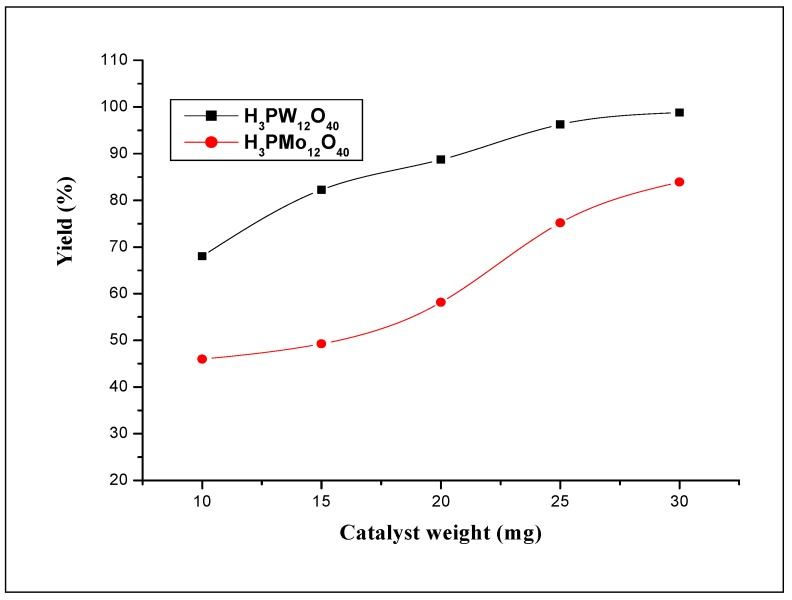
Effect of the amount of catalyst on styrene polymerization (in dichloromethane) using H_3_PW_12_O_40_ and H_3_PMo_12_O_40_ catalysts. Polymerization was carried out at 25 °C during 5 h.

Figure 5 shows the effect of the reaction time on the yields. It can be seen from the figure that the yields increased linearly for both heteropolyacids. During all the time of the polymerization, the yield of the 12-tungstophosphoric acid reaction remained higher than that obtained with 12-molybdo-phosphoric acid. The yields obtained at 5 h of reaction time with H_3_PW_12_O_40_ and H_3_PMo_12_O_40_ are 68.0% and 45.0% respectively. The average molecular weights obtained also at this reaction time are 9,250 and 5,560, respectively. As H_3_PW_12_O_40_ has stronger acidity than that of H_3_PMo_12_O_40_, these results suggest that strength acidity of the catalyst play an important role for the formation and the physical properties of polystyrene (H_3_PW_12_O_40_ > H_3_PMo_12_O_40_ [7]). Figure 6 shows the yield of polystyrene obtained for various amounts for both catalysts, H_3_PW_12_O_40_ and H_3_PMo_12_O_40_. It can be seen from this figure that both catalysts have remarkably the same tendency; however, the 12-tungsto-phosphoric acid (W as added atom) gives a higher yield than that of 12-molybdophosphoric acid (Mo as added atoms). For low catalyst amounts, one can see that the gap in the yield values between the two catalysts is high, whereas for higher amounts, the gap decreased. 

#### 2.2.3. Reactivity of W and Mo in ammonium salts

**Figure 7:**
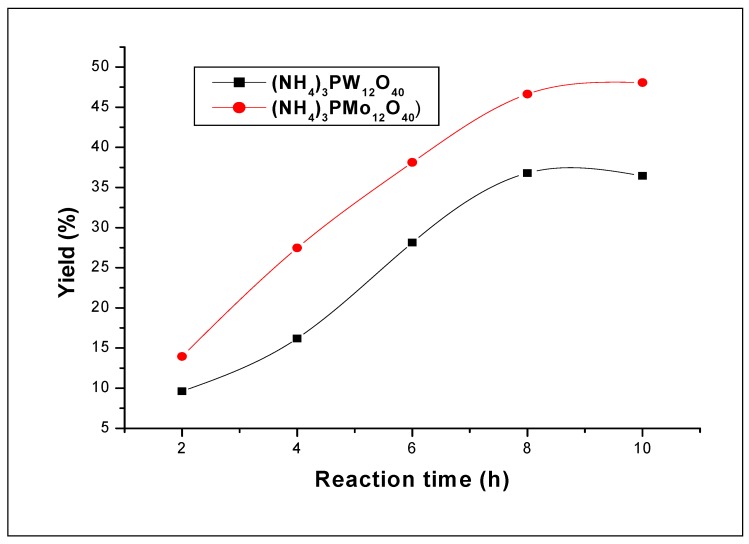
Effect of the reaction time on the styrene polymerization (in dichloromethane) using (NH_4_)_3_PW_12_O_40_ and (NH_4_)_3_PMo_12_O_40_ catalysts. Polymerization was carried out at 25 °C using 10 mg of catalyst.

The effect of the reaction time on the polymerization was shown in Figure 7. When the protons of the heteropolyacids where replaced by ammonium cations (NH_4_^+^), the obtained ammonium salts showed a dramatic decrease in the polymerization rate. In fact, no measurable yields were observed before 2 h of reaction time for both solids. Thus to obtain significant conversions, we carried out the polymerization for 12 h. Like their homologous heteropolyacids, the results showed that when the W atoms were replaced by their homologous Mo atoms, a decrease of the polymerization rate was observed. 

**Figure 8:**
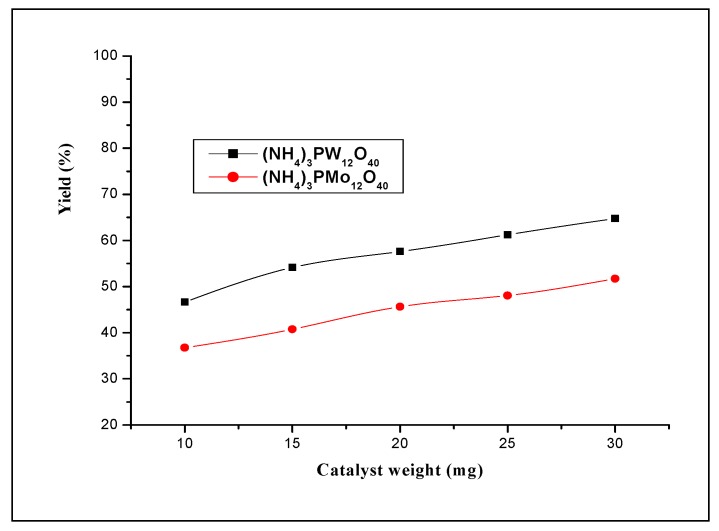
Effect of the amount of catalyst on styrene polymerization (in dichloromethane) using (NH_4_)_3_PW_12_O_40_ and (NH_4_)_3_PMo_12_O_40_ catalysts. Polymerization was carried out at 25 °C during 12 h.

The yields obtained at 12 h of reaction time with (NH_4_)_3_PW_12_O_40_ and (NH_4_)_3_PMo_12_O_40_ are 48.1% and 36.8%, respectively. The average molecular weights obtained at this reaction time are 5,657 and 4,095, respectively. As the acidity of H_3_PW_12_O_40_ is stronger than that of H_3_PMo_12_O_40_, these results suggest that strength acidity of the catalyst play an important role for the formation and the physical properties of polystyrene. The effect of the catalyst amount on the polymerization was depicted in Figure 8. The results show that for both catalysts the yields have remarkably the same tendency; however, the ammonium salt containing the W atoms presents a slight higher conversion than that containing the Mo ones.

If we take into account the yields obtained for 5 hours of polymerization with this series of catalysts; H_3_PW_12_O_40_ (68.0 %), H_3_PMo_12_O_40_ (45.0%), (NH_4_)_3_PW_12_O_40_ (33.1%), and (NH_4_)_3_PMo_12_O_40_ (22.6%) we can see that the reactivity is directly proportional to the acidity strength of the heteropolyanions (H_3_PW_12_O_40_ > H_3_PMo_12_O_40_ > (NH_4_)_3_PW_12_O_40_ > (NH_4_)_3_PMo_12_O_40_ [7]). Thus, this result indicates that strong Brønsted acidity is suitable for the cationic polymerization of styrene. 

#### 2.2.4. Effect of reaction temperature

**Figure 9:**
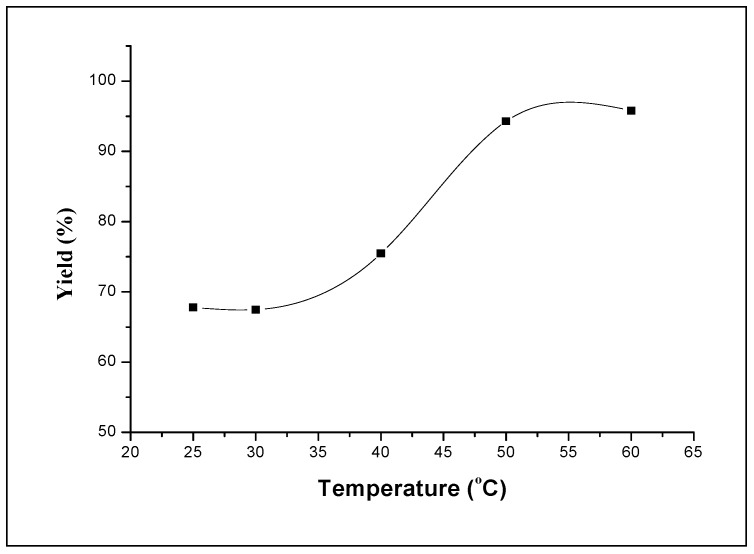
Influence of the reaction temperature on the polymerization of styrene. Polymerization was carried out in dichloromethane (St/DCM (volume ratio) = 0.4.) using 0.025 g of H_3_PW_12_O_40_ catalyst.

The effect of the polymerization temperature on the conversion was examined by keeping the composition of the St/DCM = 0.4. The results are shown in Figure 9. It can see from this figure that the rate of polymerization increases with the increase in temperature. The yield increases rapidly from 67.8% to 95.3% when the temperature increases from 25 °C to 50 °C. Then after, it remains stable at about 95.8% when the temperature increases from 50 to 60 °C. 

#### 2.2.5. Catalyst recycling

**Figure 10:**
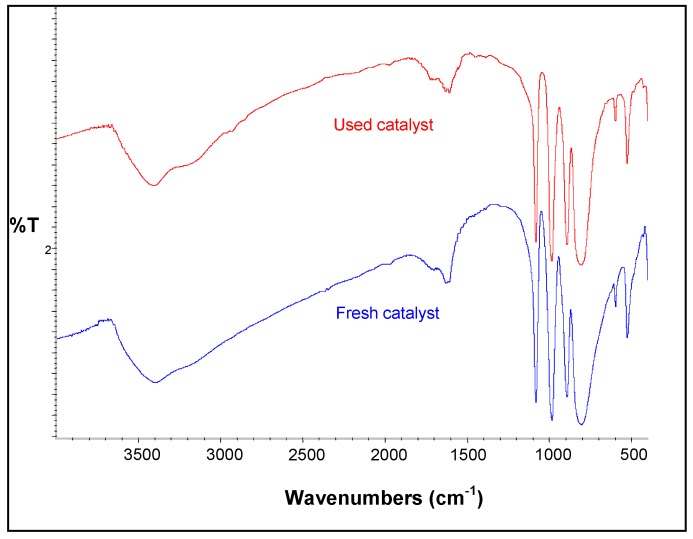
FTIR spectra of fresh and used H_3_PW_12_O_40_ catalysts.

Much effort has been devoted not only to the development of superior catalysts but also to finding ways to enable their repeated use. In order to investigate this possibility for H_3_PW_12_O_40_, this latter was separated from the reaction mixture by filtration after the reaction, regenerated by washing with cyclohexane for five times, dried at 150 over night, and used again in a fresh reaction. The IR spectra of the fresh and recycled catalyst are shown in Figure 10. It can be seen from the figure that the Keggin structure of the catalyst did not change. The regenerated catalyst was used for the recycling study under the same reaction conditions. When the reaction was carried out at 60 °C by the fresh and the used catalyst, the yield obtained is 95.8% and 72.1%, respectively. This result confirms the recyclable applicability of H_3_PW_12_O_40_ for the polymerization of styrene.

## 3. Experimental

### 3.1. Preparation of the catalysts

H_3_PW_12_O_40_·13H_2_O and H_3_PMo_12_O_40_·13H_2_O (abbreviated H_3_PW_12_O_40_ and H_3_PMo_12_O_40_) were prepared in a classical way as described in the literature [16]. (NH_4_)_3_PW_12_O_40_·4H_2_O and (NH_4_)_3_PMo_12_O_40_·4H_2_O (abbreviated (NH_4_)_3_PW_12_O_40_ and (NH_4_)_3_PMo_12_O_40_) were prepared from their homologous acids using the following procedure: the compound is precipitated by adding slowly the stoichiometric required amount of ammonium carbonate (NH_4_)_2_CO_3_ to an aqueous solution of H_3_PM_12_O_40_ (M=W; Mo). The insoluble ammonium salt is immediately precipitated. Then after the salt was filtrered off, washed with water and dried overnight at 120 °C.

### 3.2. Procedure of polymerization

The bulk polymerizations of styrene were carried out in a stirred flask at 25 °C for a definite time. Typically, a fixed amount of catalyst was added to 10 mL of styrene and 4 mL of dichloromethane under stirring. The polymerization was quenched by the addition of saturated NaOH aqueous solution. The resulting precipitated polymer was filtrated off, then dissolved in butanone to removing the catalyst, which is insoluble in butanone. The recovered polymer was precipitated into aqueous ethanol solution, filtrated off, dried at 40–50 °C under vacuum, and weighed. Then, the polymer was purified by dissolving it in chloroform and precipitation into ethanol for characterization.

#### Catalysts and polymers characterization 

The Keggin structure of the prepared heteropolyanions was checked by infrared (IR) spectroscopy. IR spectra were recorded with a GENESIS II- FTIR (4000–400 cm^-1^) infrared spectrometer as KBr pellets. ^13^C-NMR spectra were recorded on a Jeol ECLIPSE 400 MHz spectrophotometer using tetramethylsilane (TMS) as external reference. The chemical shifts are given in ppm. The glass transition temperature of the pure components was measured with a DSC (Setaram Labsys DSC 16), previously calibrated with indium, at 20 °C/min rate. The samples of 10–15 mg were preheated to 200 °C under nitrogen atmosphere and kept at that temperature for 10 min to ensure total elimination of solvent. The data were collected from the second and third scan. No degradation phenomenon of PS was observed in all thermograms. The glass transition temperature was taken as the midpoint in the heat capacity. Molecular weights were determined by measurement of viscosity. Inherent viscosity of poly(styrene) obtained were measured in toluene solution at 25 °C by using an Ubbelohde type viscometer. The viscosity average molecular weight (M_v_) was calculated by the following equation: [η]= 9.77 × 10^-3^.M_v_^0.73^ [19].

## 4. Conclusions

The polymerization of styrene in dichloremethane was performed using H_3_PW_12_O_40_, H_3_PMo_12_O_40_, (NH_4_)_3_PMo_12_O_40_, and (NH_4_)_3_PMo_12_O_40_ as catalysts. It has been found that the catalysts containing W were more reactive than those containing Mo. H_3_PW_12_O_40_ was the most effective catalyst in terms of yield and M_v_ due to its stronger Brønsted acidity. The use of H_3_PW_12_O_40_ as a heterogeneous solid acid catalyst, instead of the usual soluble inorganic acids, is a contribution to a reduction of waste. In fact, this latter can polymerize styrene under mild conditions and a simple filtration is sufficient to recover the catalyst which can be reused.
